# Fluorescence-enhanced dual signal lateral flow immunoassay for flexible and ultrasensitive detection of monkeypox virus

**DOI:** 10.1186/s12951-023-02215-4

**Published:** 2023-11-25

**Authors:** Xingsheng Yang, Xiaodan Cheng, Hongjuan Wei, Zhijie Tu, Zhen Rong, Chongwen Wang, Shengqi Wang

**Affiliations:** 1 Bioinformatics Center of AMMS, Beijing, 100850 P. R. China; 2 Beijing Key Laboratory of New Molecular Diagnosis Technologies for Infectious Diseases, Beijing, 100850 P. R. China; 3 Laboratory Medicine, Guangdong Provincial People’s Hospital, Guangdong Academy of Medical Sciences, Guangzhou, 510000 PR China

**Keywords:** Lateral flow immunoassay, Monkeypox virus, Colorimetric/fluorescent dual-mode, Dual-signal nanotag, Antigen detection

## Abstract

The outbreak of the monkeypox virus (MPXV) worldwide in 2022 highlights the need for a rapid and low-cost MPXV detection tool for effectively monitoring and controlling monkeypox disease. In this study, we developed a flexible lateral flow immunoassay (LFIA) with strong colorimetric and enhanced fluorescence dual-signal output for the rapid, on-site, and highly sensitive detection of the MPXV antigen in different scenarios. A multilayered SiO_2_-Au core dual-quantum dot (QD) shell nanocomposite (named SiO_2_-Au/DQD), which consists of a large SiO_2_ core (~ 200 nm), one layer of density-controlled gold nanoparticles (AuNPs, 20 nm), and thousands of small QDs, was fabricated instead of a traditional colorimetric nanotag (i.e., AuNPs) and a fluorescent nanotag (QD nanobead) to simultaneously provide good stability, strong colorimetric ability and superior fluorescence intensity. With the dual-signal output LFIA, we achieved the specific screening of the MPXV antigen (A29L) in 15 min, with detection limits of 0.5 and 0.0021 ng/mL for the colorimetric and fluorometric modes, respectively. Moreover, the colorimetric mode of SiO_2_-Au/DQD-LFIA exhibits the same sensitivity as the traditional AuNP- LFIA, whereas the overall sensitivity of this method on the basis of the fluorescent signal can achieve 238- and 3.3-fold improvements in sensitivity for MPXV compared with the AuNP-based LFIA and ELISA methods, respectively, indicating the powerful performance and good versatility of the dual-signal method in the point-of-care testing of the MPXV.

**Supplementary Information:**

The online version contains supplementary material available at 10.1186/s12951-023-02215-4.

## Introduction

Monkeypox virus (MPXV) is an orthopoxvirus of the poxvirus family and was first identified in monkeys for research purposes in 1958 [1, 2]. Since May 2022, the number of MPXV cases has dramatically increased globally, eventually prompting the World Health Organization (WHO) to declare the monkeypox epidemic a global health emergency [3, 4]. The MPXV is a double-stranded DNA virus that is available in two infectious forms, namely intracellular mature virus (IMV) and extracellular enveloped virus (EEV). The MPXV associates with host cells via the A29L (heparan-binding IMV surface membrane fusion protein), H3L (heparan-binding surface IMV membrane protein), and E8L (chondroitin sulfate-binding IMV surface membrane adsorption protein) proteins [5]. Infection with the MPXV can produce the zoonotic disease monkeypox (MPX) [6]. The main transmission routes of the MPXV are animal-to-person transmission and person-to-person transmission [7]. Animal-to-person transmission occurs through direct contact with the blood, body fluids, and infected sites of infected animals [8]. Person-to-person transmission is primarily via contact with the infection site and body fluids of respiratory droplets from symptomatic patients [9]. Human infection with MPX typically starts with fever, fatigue, and headache, followed by a rash that can spread to other parts of the body, with an incubation period of 5 days to 21 days [10, 11]. The early symptoms of the MPXV infection are similar to those of respiratory viruses such as severe acute respiratory syndrome coronavirus 2 (SARS-CoV-2), influenza A (FluA), influenza B (FluB), and human adenovirus (HAdV) and have a long incubation period, increasing the risk of outbreaks [12]. Meanwhile, the clinical presentation of the MPXV infection is unremarkably different from that of diseases caused by other pox viruses, especially smallpox and the varicella-zoster virus (VZV) [13, 14]. Therefore, a rapid and accurate tool for differentiating between these infectious agents is necessary for controlling the spread of outbreaks and clinical diagnosis.

Currently, polymerase chain reaction (PCR) is a common method for detecting the MPXV [9, 15]. However, the PCR method relies on professional personnel and equipment and has a long turnaround time, which cannot meet the demand for rapid on-site detection [16, 17]. Lateral flow immunoassay (LFIA), a proven POCT technique, is easy to operate, low cost, portable, and can meet the need for rapid on-site detection [18, 19]. Colloidal gold NP (AuNP)-based strips are popularly utilized in LFIA because they allow the direct interpretation of results by the naked eye [20, 21]. Nevertheless, AuNP-based LFIA has certain limitations, such as low sensitivity and inaccurate quantification due to the colorimetric signal reading limitation [22–25]. These shortcomings hinder the large-scale application of conventional LFIA in rapid and accurate inspection.

Recently, researchers achieved increased signal readability and decreased background interference using fluorescent materials such as upconversion particles or quantum dots (QDs), as labels, thereby improving the quantitative capability and sensitivity of LFIA [26–30]. Although fluorescent tags have extended LFIA to higher sensitivities, the need to employ additional instruments for fluorescent signal reading greatly limits the application of fluorescent materials in POCT. The inherent drawbacks of both the colorimetric and fluorescent LFIA methods prompted researchers to develop a colorimetric-fluorescent dual-signal nanotag with colorimetric-fluorescent properties to ensure highly flexible applications, increased colloidal/optical stability to ensure reliability in testing complex samples, and increased mobility on the test strip to ensure accurate signal readout. A series of dual-signal colorimetric-fluorescent nanotags have emerged recently, including UIO@MB, Au-QD nanorod, SiO_2_@Au@QDs, and SADs [31–34]. These nanotags have easy-to-read colorimetric signals and easy-to-quantify fluorescent signals, which can be flexibly switched according to different scenarios, expanding the application range of LFIA. SiO_2_ has attracted a considerable amount of attention due to its biocompatibility, controlled particle size, good dispersibility, and easily modified surface [35]. Our team has employed SiO_2_ as a carrier for the controlled assembly of colorimetric and optical units to improve the sensitivity and applicability of LFIA and enable the flexible monitoring of pathogens [33, 36].

Herein, we present a dual-signal LFIA sensor with an enhanced fluorescence pattern for the rapid and highly sensitive identification of the MPXV A29L protein. A29L as a surface protein of IMV has been applied to LFIA assays [37]. The innovation of the proposed dual-mode LFIA strip is reflected in the following three aspects: (i) a colorimetric-fluorescent dual-mode LFIA biosensor was proposed for the first time for the MPXV antigen detection, satisfying the rapid screening of the MPXV in resource-poor areas (colorimetry) and the highly sensitive quantitative detection in primary care institutions; (ii) a dual-signal SiO_2_-Au core dual-QD shell (DQD) nanocomposite (named Si-Au/DQD) is designed to replace traditional colorimetric tags and fluorescent materials, consisting of a monodisperse SiO_2_ core, a hybrid layer generating a colorimetric-fluorescent signal, and a fluorescence-enhanced QD shell. The high-performance Si-Au/DQD has improved fluorescence properties, effectively improving the sensitivity of the fluorescence mode of the strip; (iii) the excellent colloidal/optical stability of Si-Au/DQD enables the LFIA system to offer improved stability and sensitivity in the detection of pharyngeal swab samples. The proposed method can be directly interpreted by the naked eye or quickly measured by a portable commercial fluorescence instrument for the quantitative analysis of the MPXV. The sensitivity of the Si-Au/DQD-based LFIA colorimetric and fluorescence modes is 0.5 and 0.021 ng/mL, respectively, indicating that they are 238- and 3.3-fold more sensitive than the conventional AuNP-based LFIA and ELISA methods, respectively. Furthermore, the dual-signal sensor maintains high specificity and accuracy in pharyngeal swab spike detection, suggesting that Si-Au/DQD-based LFIA is a promising and powerful POCT platform for the flexible and accurate detection of MPXV to prevent large outbreaks of the disease.

## Experimental section

### Reagent and instrument

2-(N-morpholino) ethanesulfonic (MES), tetraethoxysilane (TEOS), branched polyethylenimine (PEI), N-(3-dimethylaminopropyl)-N′-ethylcarbodiimide hydrochloride (EDC), N-hydroxy-sulfosuccinimide (sulfo-NHS), and fetal bovine serum (FBS) were obtained from Sigma-Aldrich (St. Louis). Bovine serum albumin (BSA), ammonia (25–28 wt%), saccharose, chloroauric acid tetrahydrate (HAuCl_4_·4H_2_O), sodium azide (NaN_3_), and trisodium citrate (TSC) were supplied by Sinopharm Chemical Reagent Co. Ltd (Shanghai, China). CdSe/ZnS-MPA (red) QDs were provided by Mesolight Inc. (Suzhou, China). MPXV A29L protein (Catalog#40,891-V08E), MPXV A29L protein capture mAb (Catalog#40,891-M0017), and detection mAb (Catalog#40,891-M0027) were supplied by Sino Biological Inc. (Beijing, China). Nitrocellulose membranes (NC, CN95, and CN140) were purchased from Sartorius (Göttingen, Germany). A plastic bottom plate and sample, conjugate, and absorbent pads were obtained from Jieyi Biotechnology Co (Shanghai, China).

Transmission electron microscopy (TEM) images were recorded using a Tecnai G2 F20 microscope (Philips, Holland). The scanning electron microscopy (SEM) image of the nanobeads was taken on JSM-7001 F microscope (JEOL, Japan). The UV-vis spectra were measured with a Shimadzu 2600 spectrometer and the zeta potential data were measured using a Mastersizer 2000 (Malvern, UK). The fluorescence signals of the strip were measured using a portable fluorescence reader purchased from Suzhou Hemai Precision Instrument Co., Ltd (Suzhou, China).

### Synthesis of dual-signal Si-Au/DQD NPs

**Scheme 1:**
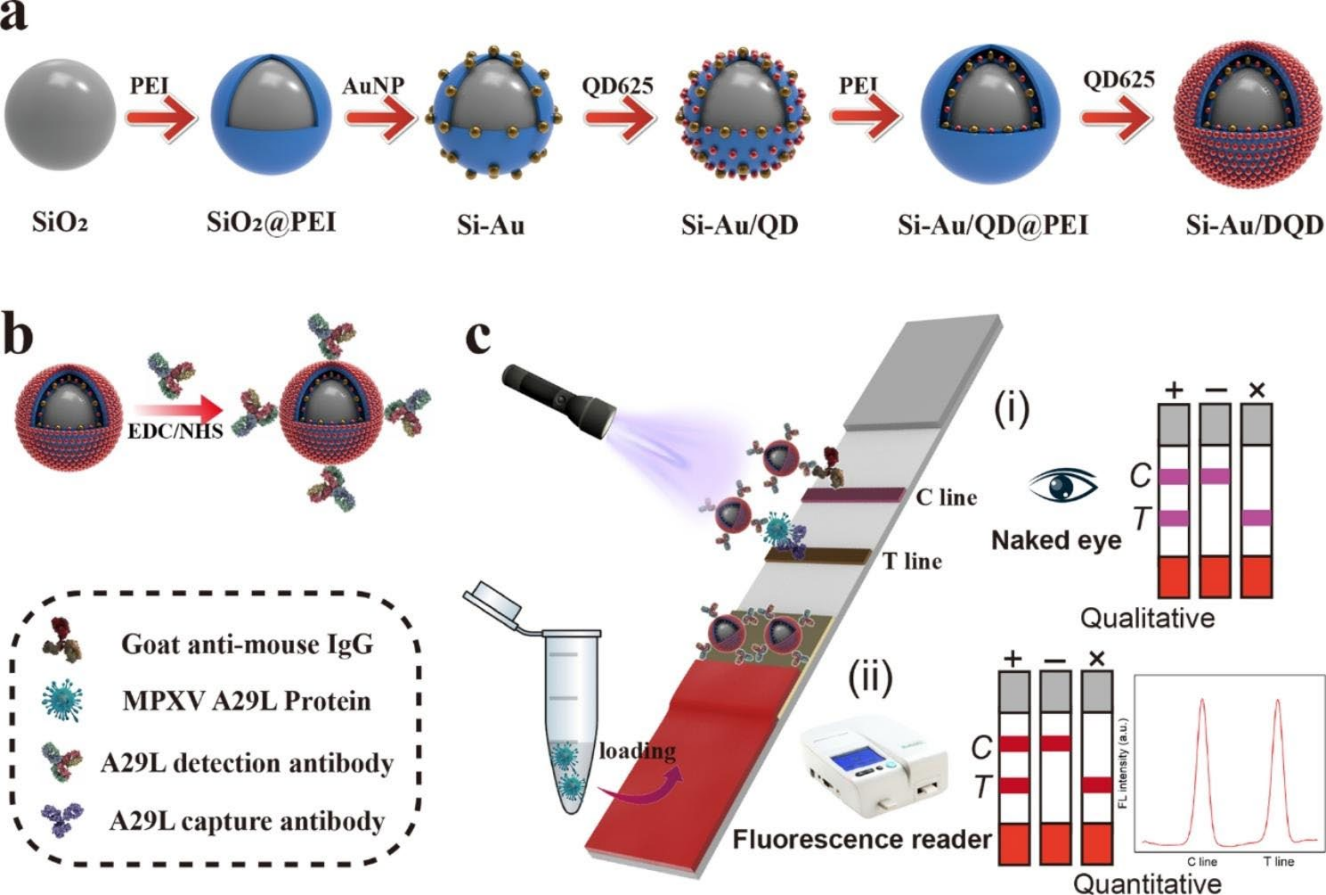
(**a**) Synthesis procedure of Si-Au/DQDs NPs, (**b**) MPXV A29L antibody coupling method, and (**c**) schematic diagram of Si-Au/DQD-based LFIA analysis of MPXV A29L protein

SiO_2_ NPs (~ 200 nm) and AuNPs (~ 20 nm) were synthesized according to a previously reported method [38, 39]. Dual-signal Si-Au/DQD NPs were synthesized by PEI-mediated electrostatic adsorption (Scheme 1a). First, 1 mL of SiO_2_ NPs was mixed with 40 mL of PEI solution (0.5 mg/mL), and SiO_2_@PEI was obtained after 40 min of sonication. The formed SiO_2_@PEI NPs were collected by centrifugation and washed with deionized water to remove the excess PEI. Second, the SiO_2_@PEI NPs were mixed with 30 mL of AuNP solution and SiO_2_-Au NPs (Si-Au) were obtained under 30 min sonication. The Si-Au NPs were gathered by centrifugation (5500 rpm, 6 min) and then scattered in 30 mL of deionized water. Third, 0.1 mL of red QDs (10 nM) was injected into the Si-Au solution. After 40 min of sonication, the negatively charged QDs were bound to the unreacted Si-Au surface sites by electrostatic adsorption, and dual-signal NPs (Si-Au/QD) with mixed shell layers of AuNPs and QDs were obtained. The Si-Au/QD NPs were centrifuged (5000 rpm, 6 min) and washed with deionized water to remove excess QD. Fourth, to improve the fluorescence performance of the NPs, another layer of QDs was adsorbed on the surface of the Si-Au/QD. Specifically, 5 mL of Si-Au/QD NPs were mixed with 40 mL of 0.5 mg/mL PEI solution to form Si-Au/QD@PEI after 40 min of sonication reaction. The resulting Si-Au/QD@PEI NPs were collected via centrifugation at 5000 rpm for 6 min and washed with deionized water to remove excess PEI. Next, 0.1 mL of 10 nM red QDs was added to 40 mL of Si-Au/QD@PEI solution and sonicated for 40 min to obtain a fluorescence-enhanced Si-Au/DQD. The Si-Au/DQD NPs were centrifuged (4500 rpm, 6 min) and resuspended in 20 ml of ethanol solution for future use.

### Preparation of Si-Au/DQD-mAb conjugates

Si-Au/DQD-mAb conjugates were prepared via carbodiimide chemistry (Scheme 1b) [40]. Briefly, 1 mL of Si-Au/DQD was centrifuged and resuspended in 50 µM of MES buffer (pH = 6.0) containing 0.5 µM of EDC and 1 µM of sulfo-NHS, and 15 min of sonication followed. The surface-activated Si-Au/DQDs were centrifuged and resuspended in 0.2 mL of 0.05% PBST, 10 µg of A29L detection antibody was added and shaken for 2 h at room temperature, and Si-Au/DQD-mAb was obtained. The Si-Au/DQD-mAb was blocked with 10% (w/v) BSA for 1 h and washed with 0.05% PBST. Finally, the Si-Au/DQD-mAb was resuspended in PBS (10 mM, pH = 7.4, 0.02% NaN_3_, 0.5% BSA, and 1% sucrose) and stored at 4 °C.

### Fabrication of the MPXV A29L LFIA strips

The structure of the LFIA strip for the detection of the MPXV A29L protein is shown in Scheme 1c. First, the Si-Au/DQD-mAb tags were sprayed onto the conjugate pad and dried at 37℃. Second, the goat anti-mouse IgG and the A29L capture antibody were immobilized in the control (C, 1.2 mg/mL, 1.0 µL/cm) and test (T, 0.9 mg/mL, 1.0 µL/cm) line areas of the NC membrane and dried at 37℃ for 12 h. Finally, the sample pad, the conjugate pad, the NC membrane, and the absorbent pad were assigned to the plastic base plate, and the card was cut into strips (3 mm) and kept in a desiccator.

### Procedure for detection of MPXV A29L protein

Briefly, the running buffer containing various concentrations of the MPXV A29L protein was loaded onto the sample pads of the strips. The experiment was repeated three times for each sample. After 15 min, the colorimetric signal of the C/T line of the strip was read directly by the naked eye. Alternatively, the fluorescence signal was recorded by a portable fluorometer for further quantitative detection.

### Pharyngeal swab simulation sample analysis

To verify the ability of the proposed method to detect real samples, we performed a simulated assay with pharyngeal swab spiked samples. A pharyngeal swab from the healthy person was dipped into the running buffer and mixed well. Different concentrations of the MPXV A29L protein standards were added to the above solution and 80 µL of the solution was added dropwise to the sample pad for detection. After 15 min, the signal of the T-line on the strip was read and recorded. The discarded strips were autoclaved and then disposed as potential biohazardous waste.

## Results and discussion

### Characterization of Si-Au/DQDs

SiO_2_ is a biocompatible monodisperse NP and has been used as a carrier for fluorescent and SERS materials in LFIA analysis. These composite NPs improve the sensitivity of LFIA, but their multi-scene application is limited by the need to equip them with additional instrumentation [41–43]. To address the need for multi-scene detection, we designed a colorimetric and fluorescence-enhanced NP for LFIA detection.

Dual-signal Si-Au/DQD NPs were synthesized using the PEI layer-by-layer (LbL) self-assembly method (Scheme 1a). The introduction of the dual-signal Si-Au/DQD NPs into the LFIA system to replace conventional tags exhibited the following features: (i) SiO_2_ as the core provides good dispersion and stability; (ii) the AuNPs and QDs adsorbed on the SiO_2_ NP surface provide good colorimetric and fluorescent signals, respectively, enabling dual-mode detection of the LFIA; (iii) the adsorbed second QD layer confers strong fluorescence to the Si-Au/DQD NPs and has more carboxylation sites, for antibody coupling, making it an ideal label for ultrasensitive LFIA.

**Fig. 1:**
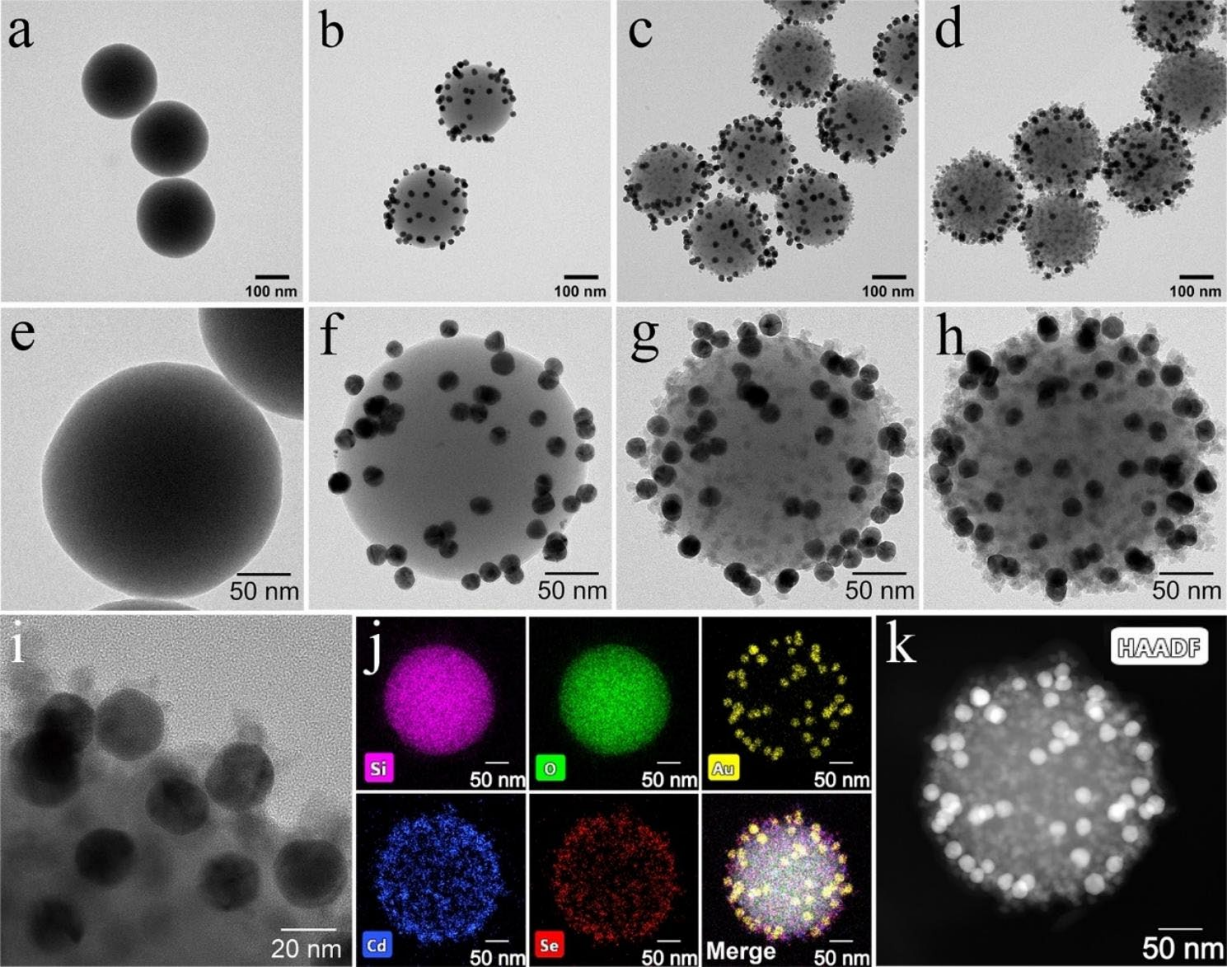
TEM images of (**a**) SiO_2_ core, (**b**) Si-Au, (**c**) Si-Au/QD, and (**d**) Si-Au/DQD NPs. High-resolution TEM images of single (**e**) SiO_2_, (**f**) Si-Au, (**g**) Si-Au/QD, and (**h**) Si-Au/DQD NP. (**i**) TEM image of Si-Au/DQD with local enlargement. (**j**) EDS elemental mapping images of a single Si-Au/DQD NP. (**k**) HADDF image of a single Si-Au/DQD NP.

The fabricated Si-Au/DQD NPs were characterized using TEM, SEM, energy-dispersive X-ray spectroscopy (EDS), and zeta potential measurements. Figure 1a-d show the TEM images of SiO_2_, Si-Au, Si-Au/QD, and Si-Au/DQD, respectively. The SiO_2_ NPs exhibited good homogeneity and dispersion (Fig. 1a), and Si-Au, Si-Au/QD, and Si-Au/DQD NPs with SiO_2_ as the core showed the same dispersion as the SiO_2_ NPs (Fig. 1b-d). Figure 1e-h show the high-resolution TEM images of SiO_2_, Si-Au, Si-Au/QD, and Si-Au/DQD, respectively. According to our previous work, many positively charged sites are formed on the surface of PEI-coated SiO_2_ NPs [12]. Therefore, the negatively charged AuNPs adhere tightly to the SiO_2_ surface by electrostatic adsorption, forming Si-Au NPs (Fig. 1f). As shown in Fig. 1f, due to the addition of a small amount of AuNPs, numerous exposed PEI sites remain on the SiO_2_ surface. The negatively charged QDs attach to the remaining exposed PEI sites through electrostatic interactions, forming Si-Au/QD NPs with mixed AuNPs and a QD layer (Fig. 1g). The co-distribution of AuNPs and QDs on the SiO_2_ surface indicates that AuNPs do not affect the subsequent adsorption of QDs. As presented in Fig. 1h, fluorescence-enhanced Si-Au/DQD NPs were obtained by repeating the adsorption process of PEI and QDs. With the adsorption of a mixed AuNP/QD layer and a QD shell, the Si-Au/DQD surface becomes rougher, providing many sites for the bio functionalization of antibodies. Figure 1i presents a partially enlarged TEM image of Si-Au/DQD. The magnified image shows that the 20 nm AuNP and 12 nm QD are closely fitted to the Si-Au/DQD surface. Figure 1j shows the EDS mapping of a single Si-Au/DQD NP. According to the distribution of the Si, O, Au, Cd, and Se elements, the Si-Au/DQD is a typical core-shell structure NP. The high-angle annular dark-field scanning TEM (HAADF) images confirm that AuNPs and QDs are distributed on the surface of the SiO_2_ (Fig. 1k). Meanwhile, the change in zeta potential also reveals the synthesis process of Si-Au/DQD NPs. As revealed in Fig. S1, the zeta potential of SiO_2_ increased sharply after PEI encapsulation, while the zeta potential decreased after the adsorption of negatively charged AuNPs and QDs, and this regular change indicates the successful construction of Si-Au/DQD NPs.

**Fig. 2:**
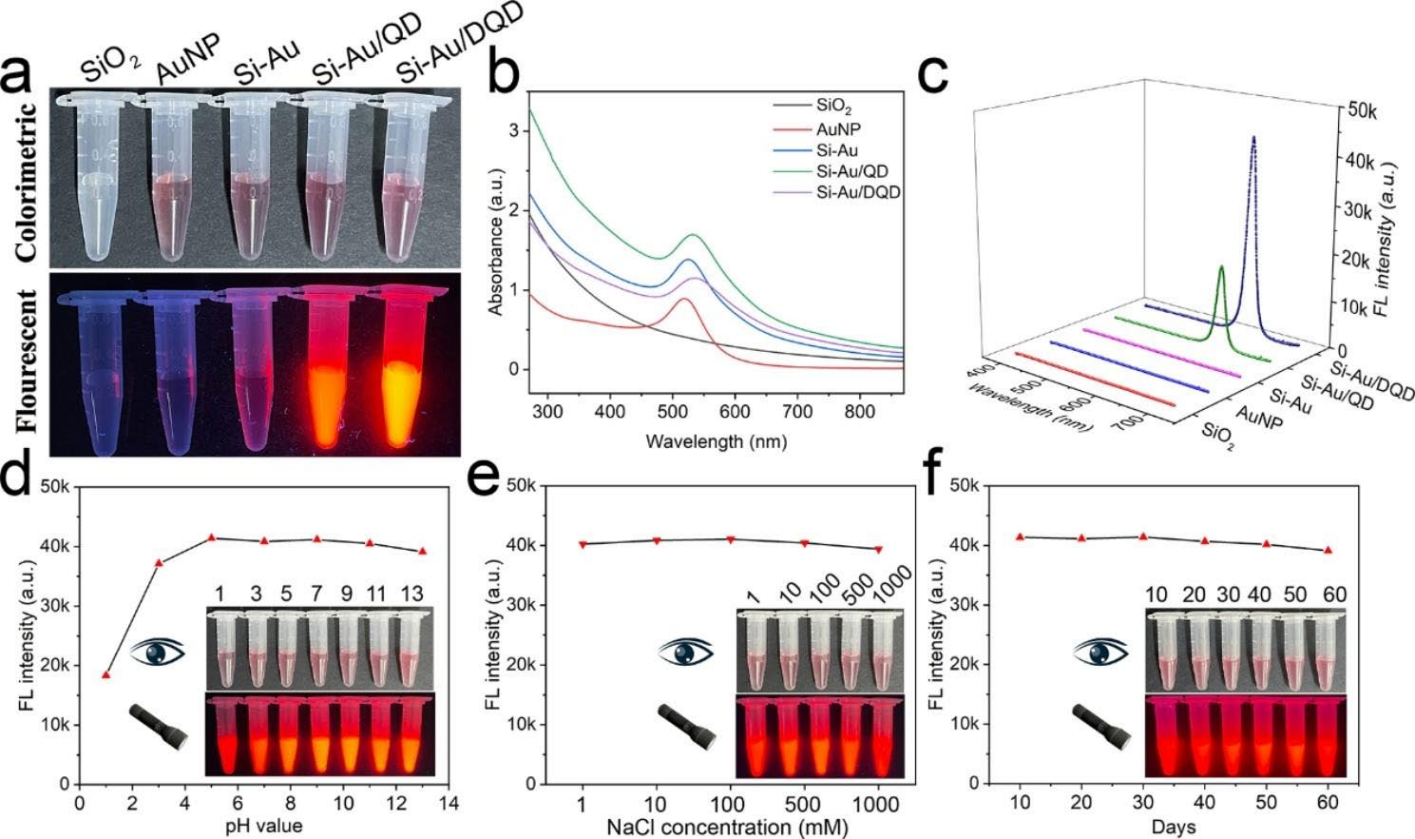
(**a**) Photographs of SiO_2_, AuNP, Si-Au, Si-Au/QD, and Si-Au/DQD under visible and UV light. (**b**) UV-vis spectra of these NPs. (**c**) Fluorescence spectra of these NPs. Stability of Si-Au/DQD under pH (**d**), salt (**e**), and time (**f**) conditions

As a promising dual-signal tag for the LFIA sensor, we systematically investigated the optical properties and colloidal stability of Si-Au/DQD. Figure 2a shows SiO_2_, AuNP, Si-Au, Si-Au/QD, and Si-Au/DQD photographs under visible and UV light. Under visible light, all the solutions, except the SiO_2_ solution, showed a purplish-red signal, because of the plasmon resonance excitation of the AuNPs. Under UV light, only the Si-Au/QD, and Si-Au/DQD solutions emitted bright fluorescence, which was caused by the fluorescence excitation of the QDs. The UV-vis spectra in Fig. 2b also confirm that the adsorption of QDs has no significant effect on the colorimetric properties of Si-Au.

The colorimetric performance of the Si-Au/QD is determined by the AuNPs adsorbed on the SiO_2_ surface, the variable QD-to-Au ratio on the SiO_2_ surface affects the fluorescence and colorimetric intensity of the dual-signal NPs. Thus, we optimized the inner layer QD-to-Au ratio. Figs. S2a-d show the TEM images of the Si-Au/QD NPs with different QD-to-Au ratios (4:1 to 1:1). The density of Au on the SiO_2_ surface increases with the Au ratio, and the intensity of the UV absorption peak increases, and the colorimetric performance is subsequently enhanced (Figs. S2e and f). However, Au and QD are co-adsorbed on the SiO_2_ surface, Au competes with QD for binding, and an increase in Au density reduces the binding of QD, weakening the Si-Au/QD fluorescence signal (Figs. S2e and g). Therefore, a balance between the colorimetric and fluorescent signals should be maintained. The Si-Au/QDs with different QD-to-Au ratios (4:1 to 1:1) were modified with antibodies and introduced into LFIA strips to detect the same MPXV samples to identify the best QD-to-Au ratios. As shown in Figs. S2 h-j, the fluorescence signal is strongest at the T line for a 4:1 ratio, but the colorimetric signal is weak. The fluorescence signal at the T-line gradually decreases and the colorimetric signal gradually increases as the ratio changes from 3:1 to 1:1, but the difference in the colorimetric signal is small. In addition, the corresponding fluorescence signal at the T-line confirms that the best results are obtained with a QD-to-Au ratio of 3:1. Therefore, the Si-Au/QD NP with a QD-to-Au ratio of 3:1 exhibits the best performance and is suitable as the core of a fluorescence-enhanced dual-signal Si-Au/DQD.

Next, we verified the fluorescence properties of each Si-Au/DQD component. Figure 2c shows no fluorescence peaks for SiO_2_, AuNP, and Si-Au, and strong fluorescence peaks for Si-Au/QD and Si-Au/DQD. The fluorescence peaks of Si-Au/DQD are stronger than those of Si-Au/QD, because more QDs are loaded on the Si-Au/DQD. As displayed in Fig. 2d-f, the fluorescence spectra, and insets under different conditions illustrate that the colorimetric properties and fluorescence intensity of Si-Au/DQD remain stable over a wide pH range (3–13) and under high salt environments and long-term storage. The Si-Au/DQD exhibited excellent stability because of the use of SiO_2_ as the core. In contrast, the ordinary AuNP (20 nm) solution was agglomerated rapidly in acidic (pH ≤ 3) solutions and highly concentrated NaCl (100–1000 mM) solutions (Fig. S3). The above results indicate that Si-Au/DQD has good colorimetric and fluorescence stability for biolabeling and is an ideal tag for LFIA.

### Construction of Si-Au/DQD-based LFIA platform

The Si-Au/DQD-based LFIA platform consists of immunochromatographic strips and a portable fluorescence reader for the qualitative or quantitative analysis of the MPXV (Scheme 1c). The constructed LFIA strip consists of four parts: a sample pad, an absorption pad, a conjugate pad coated with Si-Au/DQD-mAb, and NC membranes with C and T lines. The entire detection process was completed in one step. Briefly, the target solution was loaded onto the sample pad and the liquid migrated upward along the NC membrane through the capillary force. In the positive experiments, the A29L protein reacted preferentially with the immuno-Si-Au/DQD of the conjugated pad, and the immuno-Si-Au/DQD-A29L protein complex was subsequently captured by the immobilized secondary antibody at the T-line, forming a sandwich structure through antigen-antibody interactions. As the liquid continued to migrate, excess immuno-Si-Au/DQD was captured by the goat anti-mouse antibody in the C-line region, whereas the immuno-Si-Au/DQD was only captured by the C-line in the absence of the A29L protein in the sample solution. After a certain assay time, a qualitative result can be obtained by the visual observation of the colorimetric or fluorescence signal on the T-line. In addition, the quantitative analysis of the MPXV A29L protein was achieved using a portable fluorescence reading instrument.

**Fig. 3:**
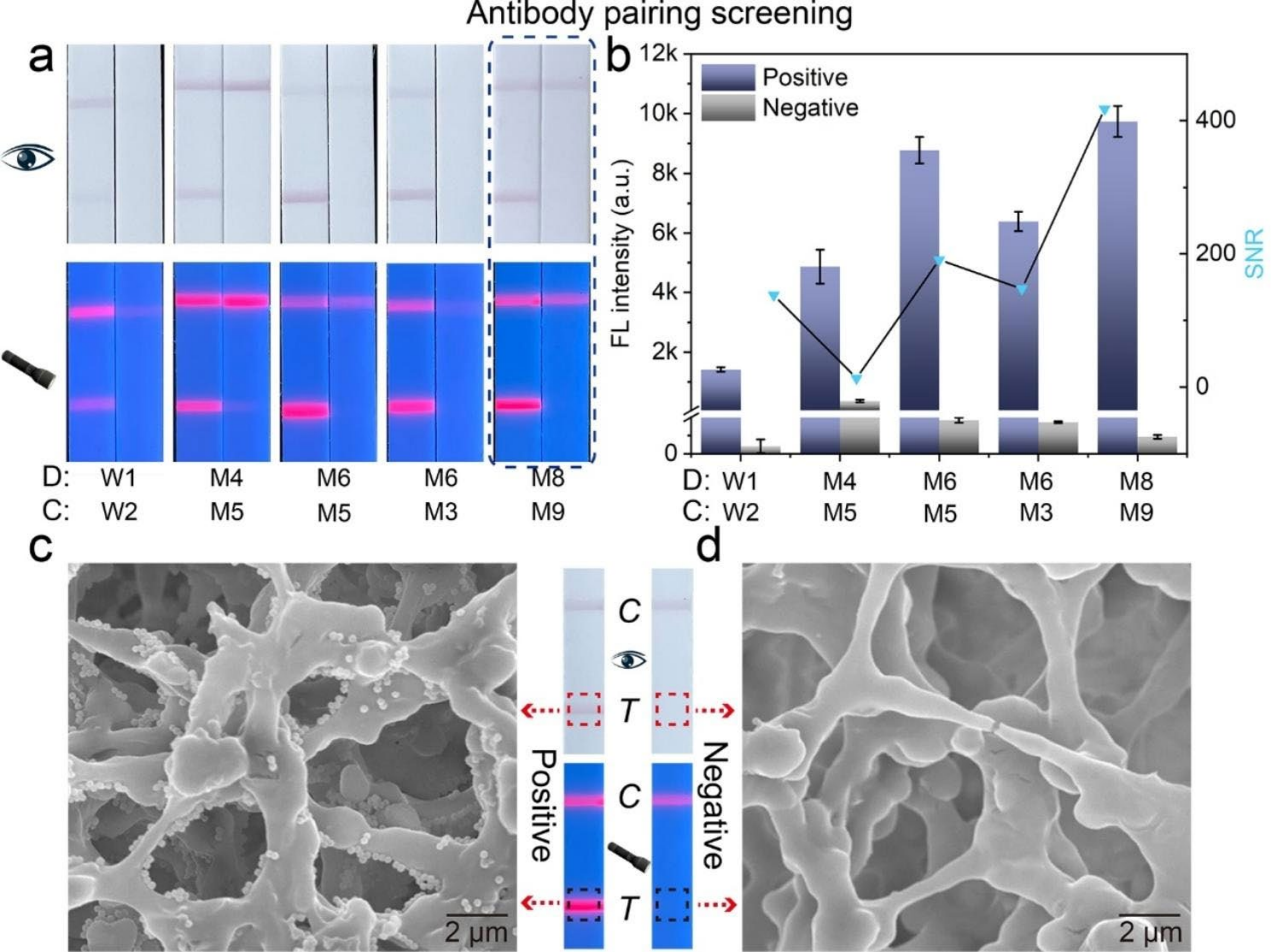
(**a**) Photographs of different antibody pairs for A29L protein detection. (**b**) SNR of different antibody pairs. SEM image from the (**c**) positive assay and (**d**) negative control

The affinity of the antibody is one of the factors that affect the LFIA sensitivity. Therefore, we screened the different antibodies and selected a pair with the best affinity for the following experiments. W1 and W2 antibodies for MPXV dection were provided by Xiamen One Clone Biotech Inc (Xiamen, China). Additional antibodies were provided by Sino Biological Inc. As shown in Fig. 3a, different antibody pairs exhibit different performance when detecting the same concentration of A29L protein (10 ng/mL). As shown by the signal-to-noise ratio (SNR) of the different pairs, the M8/M9 pair showed the best detection performance (Fig. 3b). Therefore, the M8/M9 pair was selected as the detection/capture antibody for the LFIA platform. Notably, a weak fluorescence signal on the T-line of negative sample could be recorded by the portable fluorescence instrument, which derived from the residual immuno-Si-Au/DQD labels in the running solution and can be deducted as the background noise for target detection. Meanwhile, the interior morphology of the T-line region was observed by SEM to verify the reliability of our proposed LFIA platform. As shown in Fig. 3c and d, the T-line region of the positive assay had a considerable amount of Si-Au/DQD NPs, while no Si-Au/DQD NPs were found in the T-line region of the negative control. This phenomenon indicates that the colorimetric and fluorescent signals of the strips were from the Si-Au/DQD NPs, thus demonstrating the reliability of the dual-signal LFIA platform.

The composition of the running buffer, the size of the NC membrane, and the concentration of the capture antibody are parameters that affect the performance of the LFIA assay; hence, we optimize these parameters to achieve optimal LFIA performance [44]. Fig. S4 shows that the running buffer containing 1% PBST (10 mM, pH 7.4, 1% Tween 20), 1% BSA, and 10% FBS reduced the non-specific adsorption of the Si-Au/DQD tags and maximized the SNR on the T line. As indicated in Fig. S5, the SNR of the CN140 membrane is higher than that of CN95 because the pore size of the CN140 membrane is smaller than that of CN95, and its flow rate of the solution is slower, resulting in a more adequate immune response. Finally, the influence of the antibody concentration immobilized on the T-line on LFIA was evaluated. As shown in Fig. S6, the highest SNR was obtained with the capture antibody concentration of 0.9 mg/mL. Furthermore, the optimal response time for LFIA was explored by analyzing the SNR of the T-line over a time range. As shown in Fig. S7, a reaction time of 15 min was sufficient for the quantitative detection of MPXV.

### Analytical performance of dual-signal LFIA

**Fig. 4:**
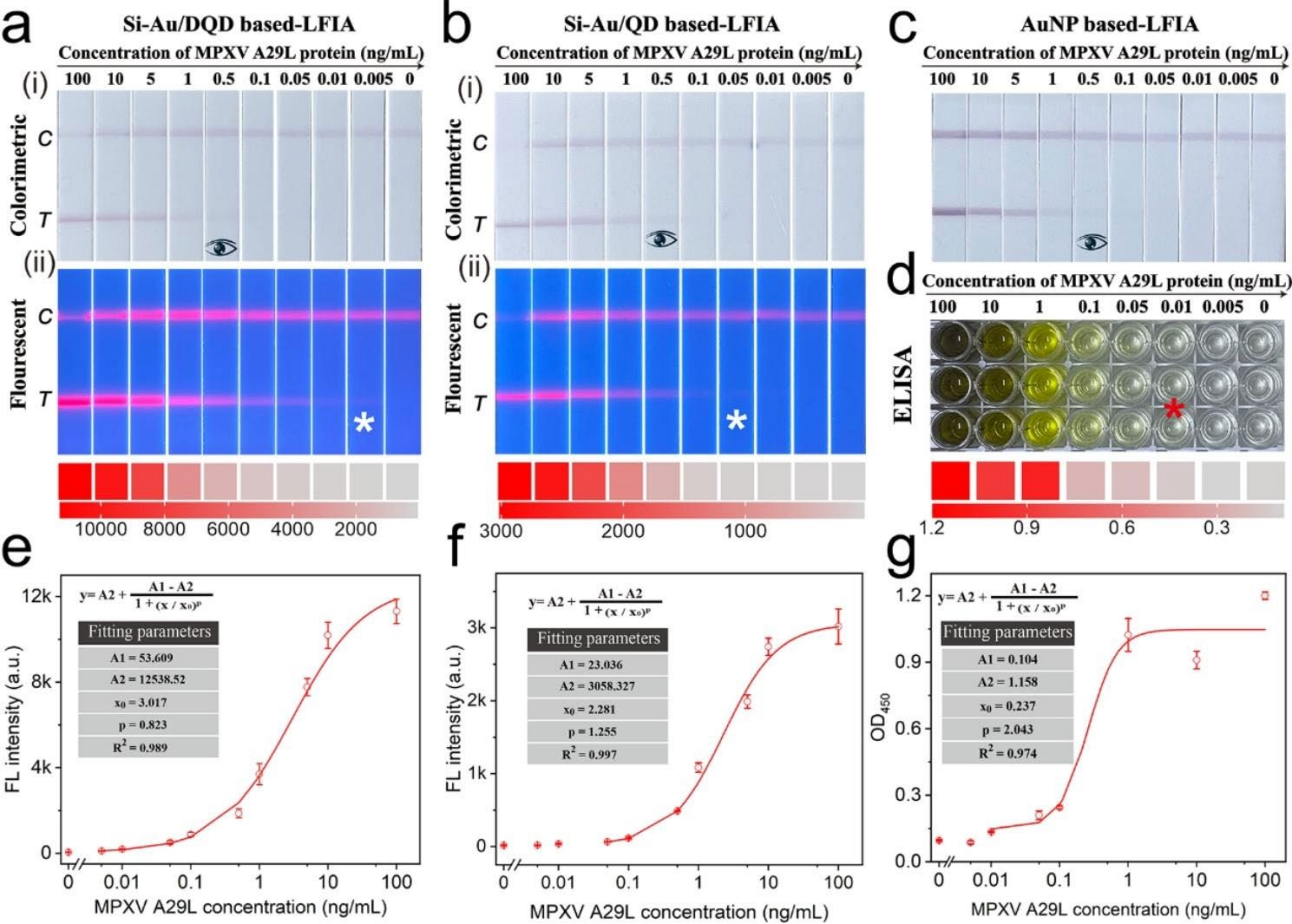
(**a**) Colorimetric (i) and fluorescence (ii) photographs of Si-Au/DQD-based LFIA strip for MPXV A29L protein detection. (**b**) Colorimetric (i) and fluorescence (ii) photographs of Si-Au/QD-based LFIA strip for MPXV A29L protein detection. (**c**) Photographs of AuNP-based LFIA strips for MPXV A29L protein detection. (**d**) Photographs of ELISA kit for MPXV A29L protein detection. (ii) Corresponding calibration curves for MPXV A29L protein detection. (**e-f**) Corresponding calibration curves of Si-Au/DQD (**e**), Si-Au/QD (**f**), and ELISA (**g**) for the detection of MPXV A29L protein assay

The performance of Si-Au/DQD-based LFIA was verified for various concentrations of the A29L protein with optimum parameters. In Fig. 4a, the photographs of the test strips under visible light and 365 nm UV light show that the samples with different concentrations of the MPXV A29L protein (0-100 ng/mL) produced different visual results. In visible light, a purple-red band appears in the region of the test strip’s C/T line, and the colorimetric signal at the T line decreased with the concentration. A faint purple-red band was observed when the concentration of the A29L protein decreased to 0.5 ng/mL, indicating a detection limit of 0.5 ng/mL in the colorimetric mode (Fig. 4ai). As shown in Fig. 4aii, the red fluorescent bands on the T line became fainter as the concentration of the A29L protein decreased, and they were positively correlated over a wide detection range of 0.005-100 ng/mL. Regardless of the mode, a reduction in the A29L protein concentration resulted in a significant decrease in the T-line signal of the test strip, verifying the feasibility of dual-signal quantitative assays. Meanwhile, the quantitative detection of the A29L protein was achieved by recording the fluorescence signal at the T-line zone and constructing the corresponding calibration curve (Fig. 4e). The obtained fluorescence signal calibration curve reveals that the fluorescence signal of the T-line is proportional to the concentration of the A29L protein in the range of 0.005 ng/mL to 100 ng/mL. The limit of detection (LOD) of the A29L protein was calculated to be 0.0021 ng/mL on the basis of the mean and triple standard deviation of the measured concentrations of the negative samples [45].

As a flexible POCT tool for MPXV detection, Si-Au/DQD-based LFIA was compared with other common immunodetection methods to evaluate its superiority. Theoretically speaking, the Si-Au/DQD tag contains a mixed AuNP/QD layer and a QD shell whereas the Si-Au/QD tag only has a mixed AuNP/QD layer, thus the Si-Au/DQD tag has more surface carboxyl groups and larger surface area for antibody modification. The maximum load capacity of antibody on the Si-Au/DQD and Si-Au/QD was investigated as shown in Fig. S8. The results suggested that Si-Au/DQD tag can load more MPXV antibody on its surface, thus possessing higher affinity to target MPXV antigen. The superior detection ability of the Si-Au/DQD labels was first verified by comparing them with monolayer Si-Au/QD tags and commercial quantum dot bead (QB) labels, using the same antibody pair. Figure 4b shows the results of the Si-Au/QD-based LFIA assay for the same A29L protein concentration. The limit of visual detection in its colorimetric mode is 0.5 ng/mL, which is the same as that of the Si-Au/DQD-based LFIA (Fig. 4bi). The fluorescence mode has a naked eye detection limit of 0.05 ng/mL, which is 10 times higher than that of the Si-Au/DQD-based LFIA (Fig. 4bii). The calibration curve constructed from the corresponding fluorescence signal shows that the LOD value of Si-Au/QD-LFIA was 11.4 times higher than that of Si-Au/QD-based LFIA (Fig. 4f). These results verified that using Si-Au/DQD with more QD layers as the fluorescent label can effectively improve the detection sensitivity of LFIA. Fig. S9a presents a TEM image of the commercially available QB, showing that the QB is slightly unstable and exhibits agglomeration. As depicted in Figs. S9b and c, the QB-based LFIA is only available in the fluorescence detection mode and has a naked eye detection limit of 0.1 ng/mL, which is 20 times lower than that of the Si-Au/DQD-LFIA. The low sensitivity is caused by fluorescence attenuation due to QB agglomeration. In addition, we carried out a comparison with the commonly used AuNP-based LFIA method to verify the colorimetric performance of the Si-Au/DQD-based LFIA. Figure 4c shows that the naked eye detection limit of the AuNP-based LFIA is 0.5 ng/mL under the same antibody pairing, which is on par with the colorimetric mode of the Si-Au/DQD-based LFIA. However, in the fluorescence mode, the Si-Au/DQD-based LFIA exhibited a 238-fold decrease in LOD compared with the conventional AuNP-based LFIA. Subsequently, we performed a comparison with the Si-Au/DQD-LFIA using a commercial ELISA kit purchased from Beijing Sino Biological Inc (Catalog# KIT40891). Figure 4d shows the results of the ELISA analysis of the A29L protein. The OD_450_ was recorded with a microplate reader to construct a calibration curve for the detection of the MPXV A29L protein. As shown in Fig. 4g, the LOD of the ELISA method for detecting the A29L protein was determined to be 7.1 pg/mL, which based on the IUPAC protocol. This result indicated the LOD of ELISA is about 3.3-fold higher than that of the Si-Au/DQD-based LFIA.

Furthermore, the commercial AuNP-based LFIA can only detect 0.5 ng/mL of the target, which is 238 times higher than that of the Si-Au/DQD-LFIA (Fig. S10). The linear range of four detection methods (Si-Au/DQD-LFIA, Si-Au/QD-LFIA, AuNP-based LFIA and ELISA) were shown in Fig. S11. In addition, the main performance indexes of the four methods for for MPXV antigen detection were summarized in Table S1. These results fully demonstrate the flexibility and highly sensitive detection capability of dual-signal LFIA. Thus, the Si-Au/DQD-based dual-mode LFIA enables the flexible readout of signals and proves its suitability for POCT in resource-limited situations.

### Reproducibility and specificity of dual-signal LFIA

**Fig. 5:**
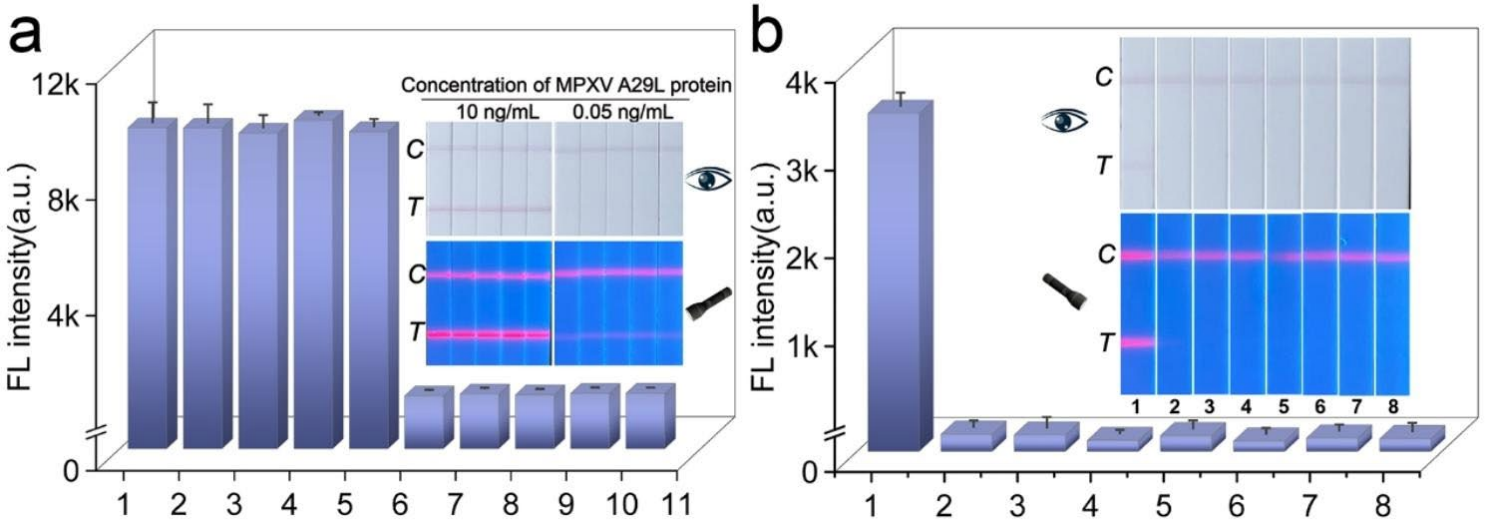
(**a**) Reproducibility of Si-Au/DQD-based LFIA. (**b**) Specificity of Si-Au/DQD-based LFIA. The numbers 1–8 at the bottom of the strip represent the detection targets of MPXV (1ng/mL), VCAV (100 ng/mL), CPXV (100 ng/mL), VZV (100 ng/mL), SARS-CoV-2 (10^6^ pfu/mL), FluA (10^6^ pfu/mL), FluB (10^6^ pfu/mL), and HAdV (10^6^ pfu/mL)

The reproducibility and specificity of the Si-Au/DQD-based LFIA were investigated to assess its feasibility in field applications. The reproducibility of the Si-Au/DQD-LFIA was verified by detecting the intermediate and low concentrations of the A29L protein. As shown in Fig. 5a, both colorimetric and fluorescence modes showed good fluorescence signal reproducibility on the T-line when detecting the intermediate concentrations of the target. In detecting low concentrations of targets, only the T-line of the fluorescence mode showed signals and exhibited good reproducibility, indicating that the proposed dual-signal LFIA has good reproducibility in different target concentrations. In addition, the specificity of Si-Au/DQD-LFIA was evaluated against other orthopoxviruses and respiratory viruses, including the vaccinia virus (VCAV, A27), the cowpox virus (CPXV, 162), VZV, FluA, FluB, SARS-CoV-2, and HAdV [46, 47]. Figure 5b shows that only the test strip with the A29L protein detection target showed distinct colorimetric and fluorescent bands in the T-line zone, and none of the test strips for the other targets showed colorimetric and fluorescent lines, demonstrating the high specificity of the method for the A29L protein.

### Application in actual samples

**Fig. 6:**
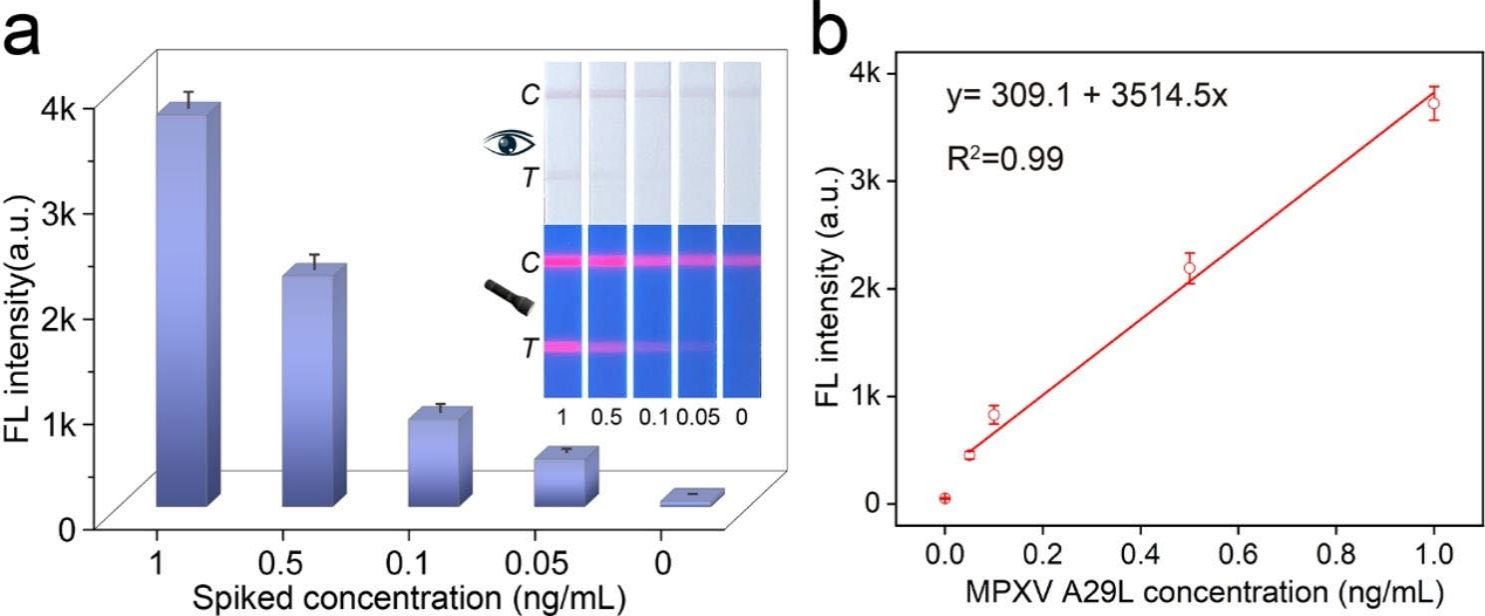
(**a**) Colorimetric/fluorescent images of the strips and the corresponding T-line signal intensities for MPXV detection (1-0.05 ng/mL). (**b**) The linear relationship of the fluorescence signals with MPXV concentrations

We further evaluated the feasibility of Si-Au/DQD-based LFIA by analyzing the MPXV A29L protein spiked pharyngeal swab samples from healthy individuals due to the lack of clinical samples. Recovery experiments were performed on pharyngeal swab samples spiked with concentrations of 1, 0.5, 0.1, and 0.05 ng/mL and are summarised in Fig. 6; Table 1. For high concentrations, the samples can be quantified directly with the naked eye, and for low concentrations, they can be measured with the aid of a UV lamp or a portable fluorescence reader (Fig. 6). Furthermore, we calculated the recovery rate in the fluorescence mode. As listed in Table 1, the spiked recoveries in the fluorescence mode were 88.8-111.5% and the coefficients of variation were 4.22-10.28%, which are consistent with the requirements for quantitative assay of the actual samples.

**Table 1:** Recovery efficiency of MPXV A29L protein detected in pharyngeal swab samples Si-Au/DQD -based LFIA.

*Spiked concentration* *(ng/mL)*	*Found concentration* *(ng/mL)*	*Recovery (%)*	*CV (%)*
1	1.039	103.9	4.22
0.5	0.444	88.8	6.48
0.1	0.112	111.5	10.28
0.05	0.048	96.0	7.73

## Conclusions

In conclusion, we proposed a dual-signal output LFIA tool for the rapid and flexible detection of MPXV in complex clinical samples. Dual-signal Si-Au/DQD tags were easily prepared through a PEI-mediated LbL assembly method and then introduced into the LFIA system to improve the performance of current immunochromatography methods. The established assay can achieve qualitative sensing and quantitative analysis of the MPXV within 15 min, with LODs of 0.5 and 0.021 ng/mL in the colorimetric and fluorescence modes, respectively.

The detailed comparative experiments revealed that the sensitivity of the proposed method is 238- and 3.3-fold higher than those of the conventional AuNP-based LFIA method and the ELISA kit, respectively. Meanwhile, the platform is sensitive, specific, and reproducible in real pharyngeal swab samples, validating its potential in clinical diagnosis. Furthermore, the platform that integrates colorimetric and fluorescence modes has a wide range of applications and can be switched flexibly according to the needs of the scenarios. We believe that this method will play an important role in the field of rapid MPXV detection.

### Electronic supplementary material

Below is the link to the electronic supplementary material.

Supplementary Material 1

## Data Availability

All data in support of the results of this study can be obtained from the Supplementary Information.
