# Supplementary material for: Fluorescence-enhanced dual signal lateral flow immunoassay for flexible and ultrasensitive detection of monkeypox virus
Source: J Nanobiotechnology. 2023 Nov 25;21:450. doi: 10.1186/s12951-023-02215-4 (PMC10675944; doi:10.1186/s12951-023-02215-4)
Supplement: Supplementary file 1 — Supplementary Material 1 [file 12951_2023_2215_MOESM1_ESM.docx]

Supplementary Information

**Fluorescence-enhanced dual signal lateral flow immunoassay for flexible and ultrasensitive detection of monkeypox virus**

Xingsheng Yang^a,b^, Xiaodan Cheng^a,b^, Hongjuan Wei^a,b^, Zhijie Tu^a,b^, Zhen Rong^a,b,^*, Chongwen Wang^a,c,^*, Shengqi Wang^a,b,^*

^a^ Bioinformatics Center of AMMS, Beijing, 100850, P. R. China

^b^ Beijing Key Laboratory of New Molecular Diagnosis Technologies for Infectious Diseases, Beijing, 100850, P. R. China

*Corresponding author

Email: [rongzhen0525@sina.com](mailto:rongzhen0525@sina.com) (Zhen Rong),

Email: wangchongwen1987@126.com (Chongwen Wang),

Email: sqwang@bmi.ac.cn (Shengqi Wang),

**S1 Preparation of AuNP-based LFIA**

First, we prepared 40 nm Au NPs using a conventional trisodium citrate reduction method. Second, the pH of 15 μg of MPXV A29L antibody was adjusted to 9 with 0.2 M K_2_CO_3_ and incubated with 1 mL 40 nm AuNP (pH 8-9) for 15 min. Then, 100 μL of 10% BSA solution (w/v) was added to block the unreacted sites of AuNPs. The as-prepared immuno-AuNPs were separated by centrifugation (4000 rpm, 6 min), and resuspended with 200 µL of a storage solution (10 mM PB solution containing 1% BSA (w/v), 0.1% PVP (w/v), 10% sucrose (w/v), and 0.05% Tween-20 (v/v). Finally, the immuno-AuNPs was bound to the conjugate pad by using the vacuum freeze dryer LGJ-10C. All the components were assembled on a plastic backing card, and the LFIA card was cut into 3 mm strips for further use.

**S2 Preparation of QB-Based LFIA**

The conjugation of commercial QB and antibodies was conducted via carbodiimide chemistry. First, 25 μL of QB was added in 25 μL MES buffer (100 mM, pH 6.0), and then mixed with 2 μL of EDC (10 mM) and 4 μL of sulfo-NHS (10 mM). After activation for 15 min, the mixture was centrifuged to remove the excess activators (EDC/sulfo-NHS) and dispersed in 25 μL PBS buffer (10 mM, pH 7.4). Then, the activated QB solution was incubated with 10 μg of MPXV A29L antibody for 1 h at room temperature, followed by surface blocking with 100 μL 10% BSA solution (w/v) for 30 min. The antibody-conjugated QBs were separated by centrifugation (9000 rpm, 6 min), and resuspended with 25 µL of a storage solution (10 mM PBS containing 1 mg BSA (w/v), 0.5% sucrose (w/v), 0.02% NaN3 (w/v)). The immuno-QB was bound to the conjugate pad by using the vacuum freeze dryer LGJ-10C. All the components were assembled on a plastic backing card, and the LFIA card was cut into 3 mm strips for further use.


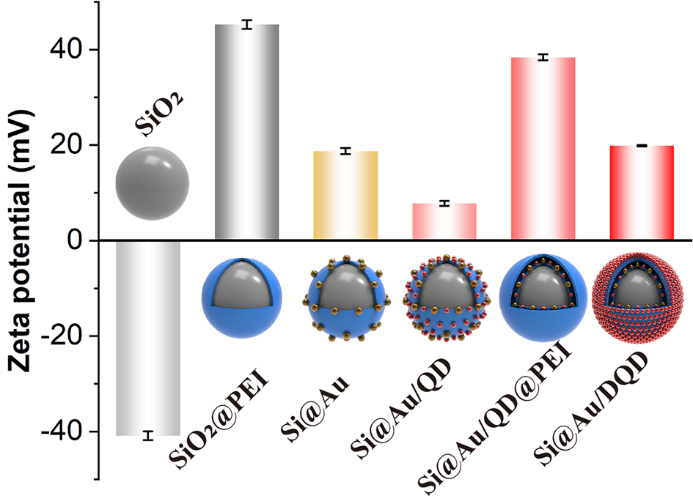


**Fig. S1.** Zeta potential of each phase of Si-Au/DQD.


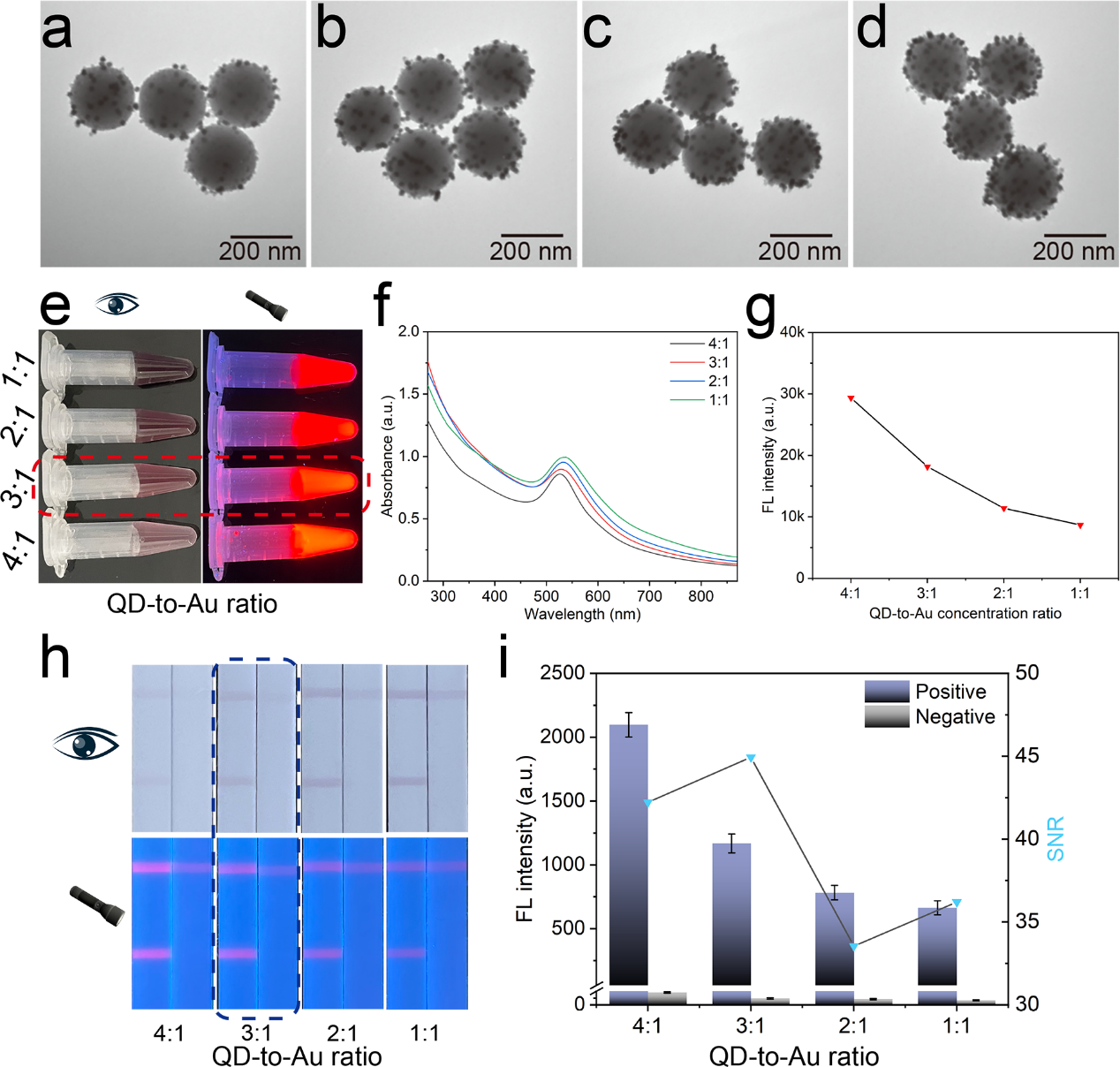


**Fig. S2.** TEM image of Si-Au/QD NPs with different QD-to-Au ratio, (a) 4:1, (b) 3:1, (c) 2:1, (d) 1:1. (e) Photographs of Si-Au/QD under UV and daylight. (f) UV-vis spectra and (g) fluorescence spectra of Si-Au/QD. (h) Photographs of Si-Au/QD-based LIFA strips under UV and daylight. (i) The corresponding fluorescence signal of the strips.


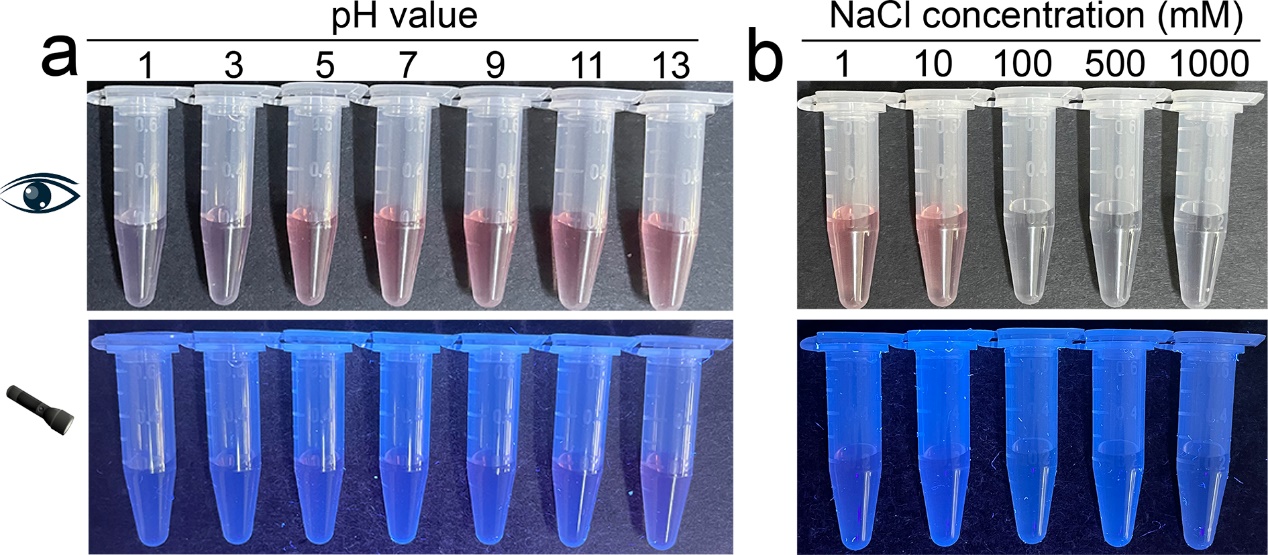


**Fig. S3.** Stability of AuNP in pH (a) and salt (b) solutions.


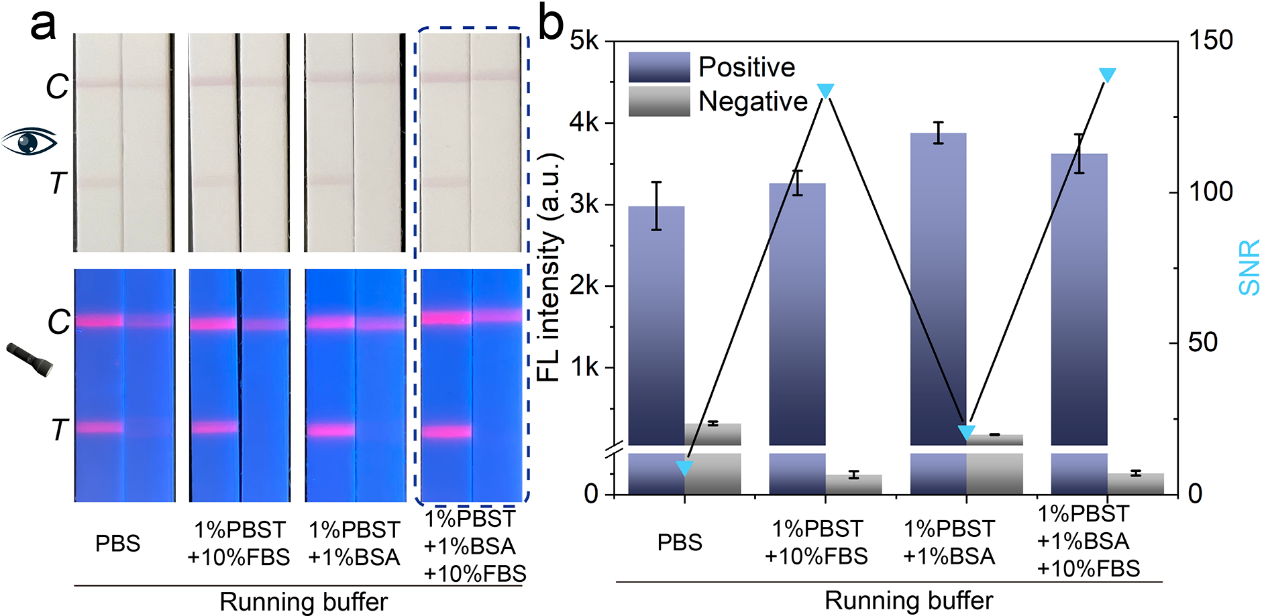


**Fig. S4.** Optimization of the running buffer. (a) Photographs of the strips under visible light and UV 365 light, (b) corresponding to the fluorescent signal.


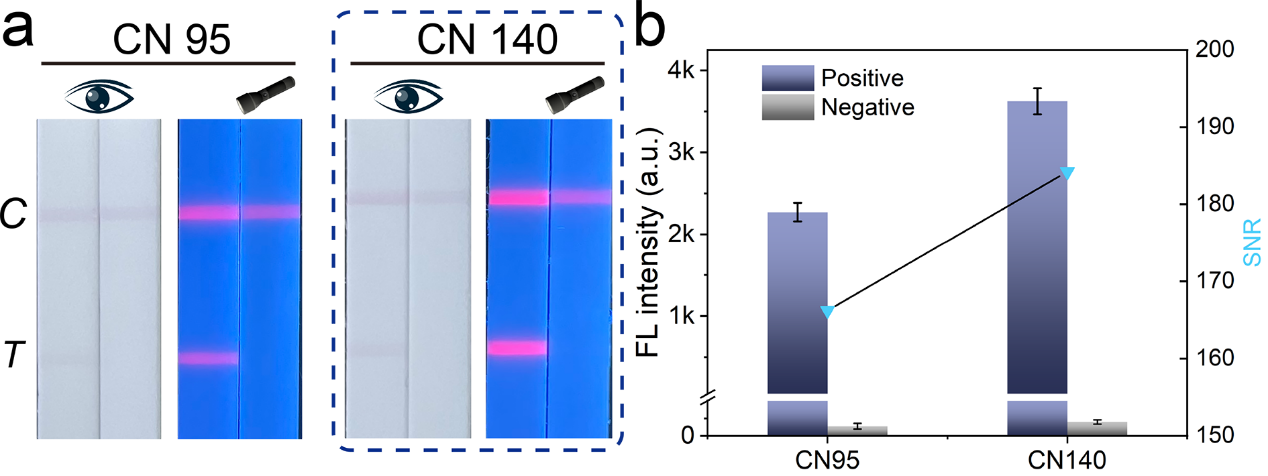


**Fig. S5.** Optimization of the NC membrane. (a) Photographs of the strips under visible light and UV 365 light, (b) corresponding to the fluorescent signal.


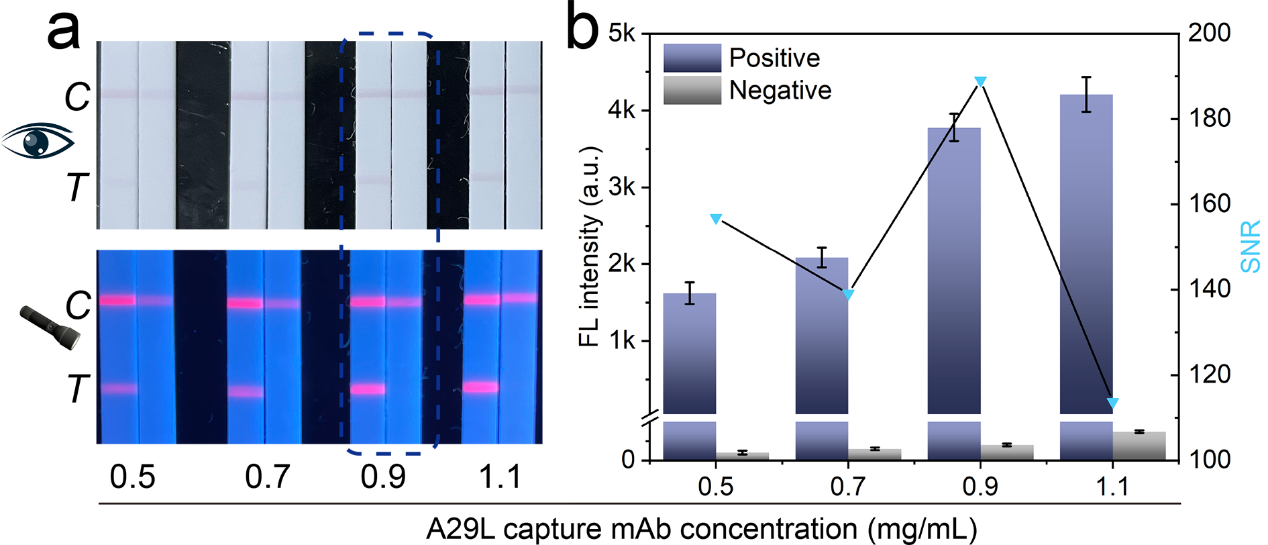


**Fig. S6.** Optimization of capture antibody concentration on T-line. (a) Photographs of the strips under visible light and UV 365 light, (b) corresponding to the fluorescent signal.


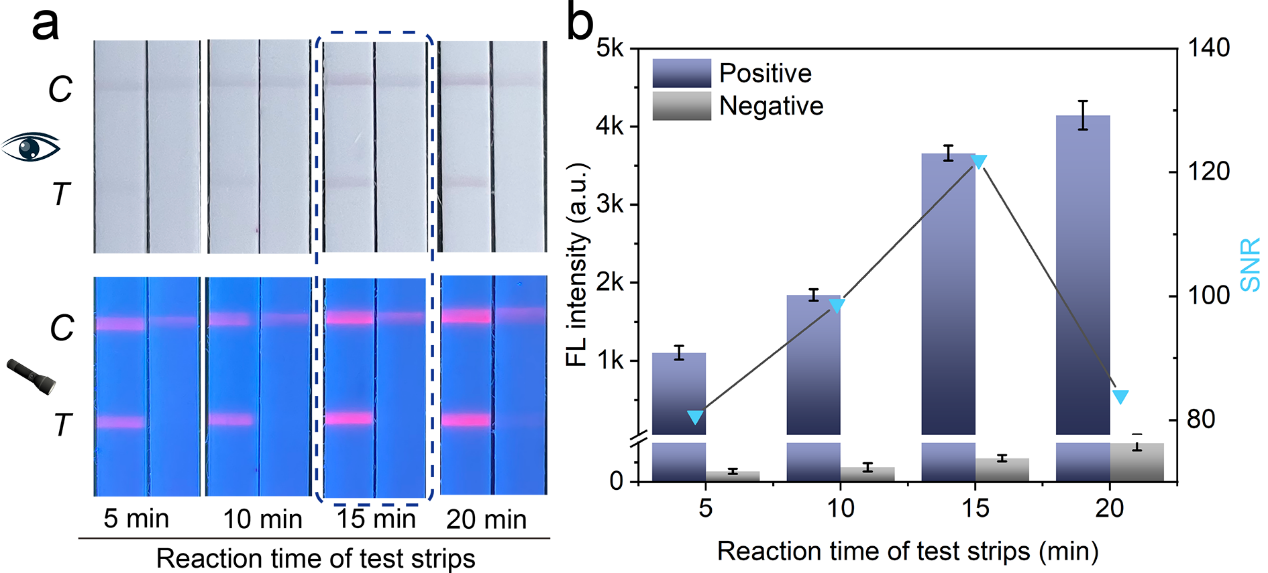


**Fig. S7.** Optimization of reaction time. (a) Photographs of the strips under visible light and UV 365 light, (b) corresponding to the fluorescent signal.


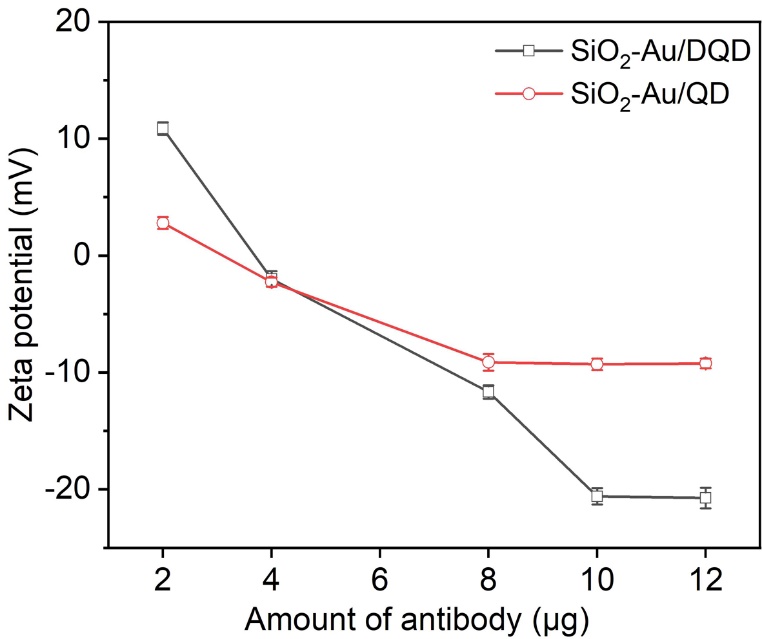


**Fig. S8.** Zeta potential of fabricated nanotags versus the number of surface-modified A29L antibodies.

The maximum load capacity of antibody on the Si-Au/DQD and Si-Au/QD was investigated by incubating different amount of MPXV antibody (2-12 μg) with 1 mg of Si-Au/DQD and Si-Au/QD, respectively. As shown in Fig. S8, the zeta potential values of immuno-Si-Au/DQD and immuno-Si-Au/QD tags remained stable by conjugating with 10 and 8 μg of antibody, respectively, indicating the amounts of antibodies modifed onto the tags reached saturation. These results revealed that Si-Au/DQD tags have larger surface area for antibody coupling than Si-Au/QD.


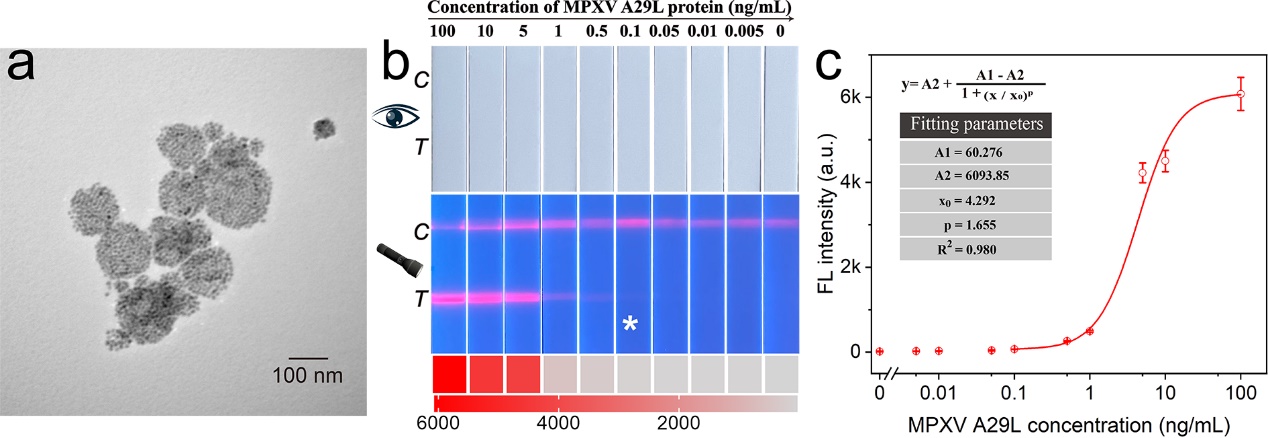


**Fig. S9.** (a) TEM image of a commercial QB. (b) Photographs of commercial QB-based LFIA strip for MPXV A29L protein detection. (b) Corresponding calibration curves for MPXV A29L protein detection.


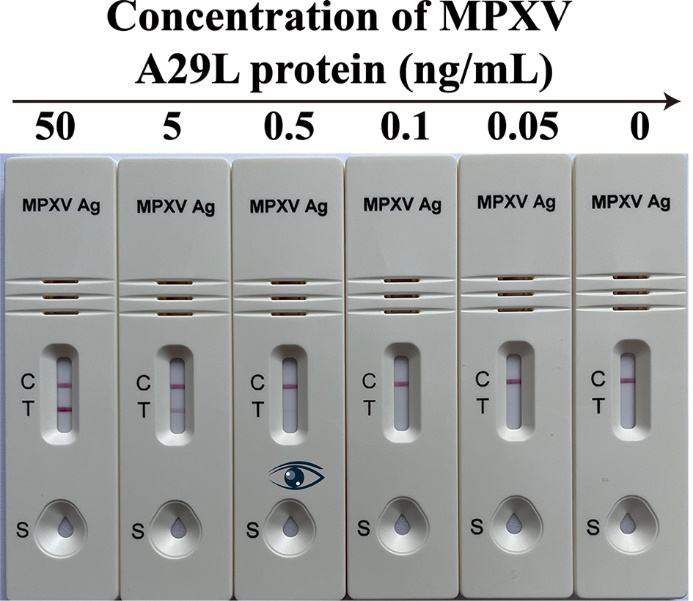


**Fig. S10.** Results of commercial AuNP-based LFIA strips for the detection of MPXV A29L protein.


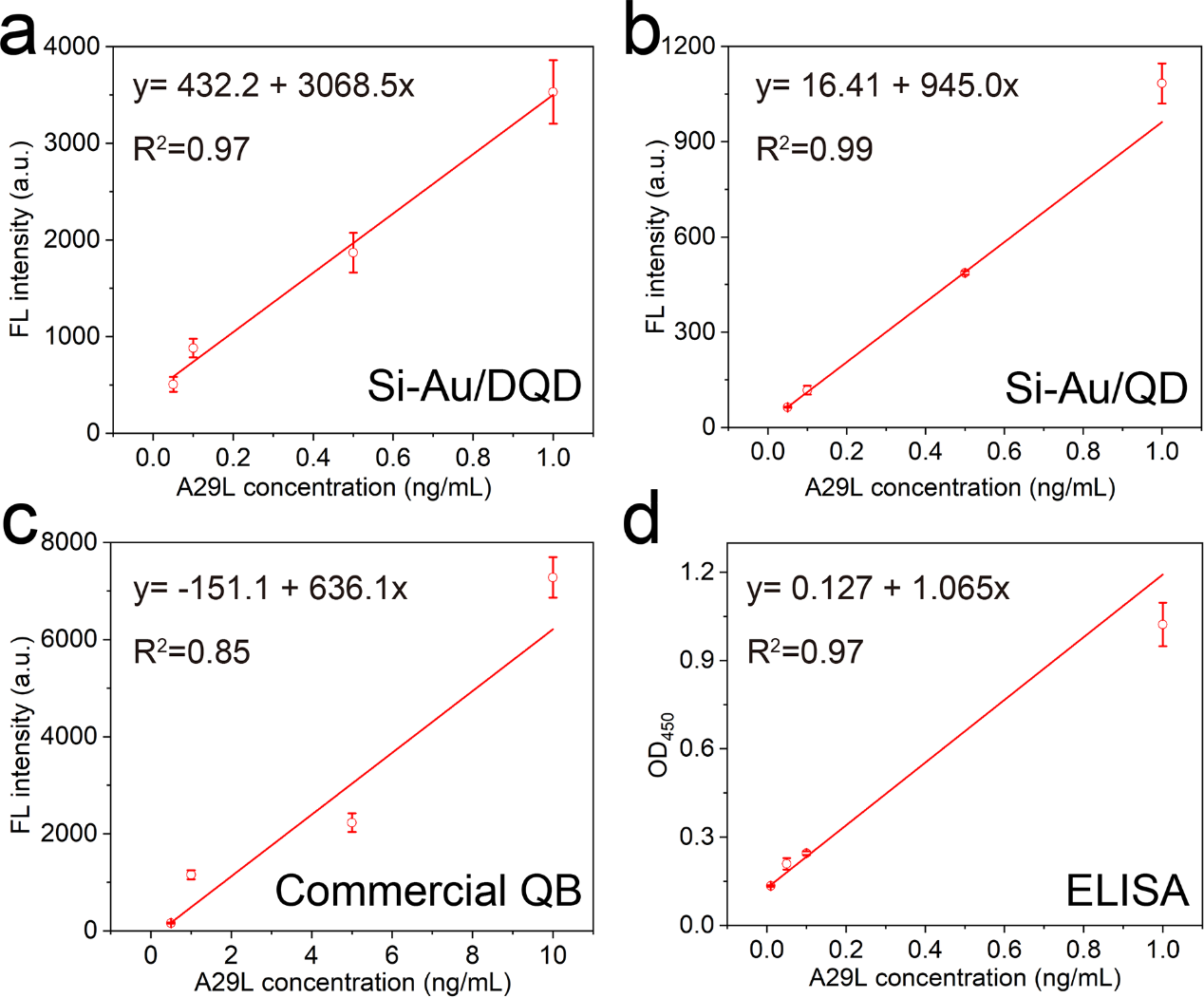


**Fig. S11.** Linear response of the (a) Si-Au/DQD-LFIA, (b) Si-Au/QD-LFIA, (c) commercial QB-LFIA and (d) ELISA for MPXV detection.

**Table S1.** The comparison of different detection methods.

| ***Detection method*** | ***Linear range (ng/mL)*** | ***LOD (ng/mL)*** |
| --- | --- | --- |
| Si-Au/DQD-LFIA | 0.05-1 | 0.0021 |
| Si-Au/QD-LFIA | 0.05-1 | 0.024 |
| Commercial QB-LFIA | 0.5-10 | 0.1 |
| ELISA | 0.01-1 | 0.0071 |
